# Comparation and evaluation of the accuracy of the sulcus localization method to establish the medial patellofemoral ligament femoral tunnel: a cadaveric and clinical study

**DOI:** 10.1186/s12891-019-2439-x

**Published:** 2019-02-07

**Authors:** Xuancheng Zhang, Guoming Xie, Chengyuan Zhang, Zhaoyi Fang, Jinzhong Zhao, Xiaoqiao Huangfu

**Affiliations:** Department of Sports Medicine, Shanghai Jiao Tong University Affiliated Sixth People’s Hospital, 600 Yishan Road, Shanghai, 200233 China

**Keywords:** Medial patellofemoral ligament, Femoral tunnel, Sulcus, Localization method

## Abstract

**Background:**

In anatomic medial patellofemoral ligament (MPFL) reconstruction, malpositioning of the MPFL femoral tunnel is common. A palpable sulcus reportedly exists at the anatomic femoral attachment of the MPFL. The present study aimed to investigate the accuracy of the sulcus localization method to establish the MPFL femoral tunnel.

**Methods:**

A cadaveric study was first done on 12 knees to evaluate the accuracy of the sulcus localization method to establish the entry points of the MPFL femoral tunnel in comparison with the midpoint and fluoroscopic localization methods. The center of the native MPFL femoral attachment was served as the reference in the cadaveric study. A clinical study was then performed to further evaluate the accuracy of the sulcus localization method in 53 patients (60 knees). Schöttle’s point was served as the reference in the clinical study. Femoral tunnel placement was defined as accurate when it was less than 5 mm from Schöttle’s point. In both the cadaveric and clinical studies, MPFL femoral tunnel placement was assessed on postoperative reconstructed three-dimensional computed tomography images. In the cadaveric study, the accuracy of different localization methods was compared using analysis of variance.

**Results:**

In the cadaveric study, the mean distances from the native MPFL attachment to the femoral tunnel entry point were 4.2 ± 1.0 mm (range 2.4–5.6 mm), 4.4 ± 1.4 mm (range 1.8–6.6 mm) and 2.9 ± 0.8 mm (range 1.9–4.4 mm) using the midpoint, fluoroscopic, and sulcus localization methods, respectively; this distance significantly differed between the midpoint and sulcus localization methods, and between the fluoroscopic and sulcus localization methods (*p* ≤ .05). While there were no significant differences between the midpoint and fluoroscopic localization methods (n.s.). In the clinical study, the mean distance between the femoral tunnel and Schöttle’s point was 3.5 ± 1.5 mm (range 0.4–6.1 mm), with accurate tunnel placement achieved in 49 of 60 cases (82%).

**Conclusion:**

The sulcus localization method can accurately guide MPFL femoral tunnel placement. This method might be useful for orthopedic surgeons.

**Level of evidence:**

IV

## Background

The prevalence of medial patellofemoral ligament (MPFL) injury reportedly ranges from 95 to 100% in patellar dislocation cases [1]. Recent research into the MPFL anatomy and kinematics has increased the interest in performing anatomical MPFL reconstruction to restore the natural behavior of the patella during knee flexion [1–6]. A malpositioned MPFL femoral tunnel reportedly leads to patellofemoral joint disturbance and recurrence of patellar dislocation [3, 7–11]. Although various localization methods that were proposed based on radiographic and gross dissection studies have described the guidance of MPFL femoral tunnel placement [1, 2, 4, 7, 8, 12–18], malpositioning of the MPFL femoral tunnel is still common [7, 8, 11, 19].

The two methods commonly used to position the MPFL femoral tunnel in current clinical practice are midpoint localization in which the MPFL femoral tunnel entry point is made at the midpoint between the adductor tubercle (AT) and the medial epicondyle (ME) [7, 8, 12], and fluoroscopic localization in which Schöttle’s point is located via repeated fluoroscopy [4, 8]. Recent research has described a sulcus at the MPFL femoral attachment between the AT, ME, and gastrocnemius tubercle (GT) [5, 20, 21]. However, there is very little published data concerning the practicability and accuracy of using sulcus localization to establish the best position of the MPFL femoral tunnel. Our hypothesis was that sulcus localization could be used to achieve accurate placement of the MPFL femoral tunnel.

## Methods

The present study was composed of two parts: a cadaveric study, and a clinical study. The cadaveric study was performed between May 2017 and June 2017 to compare the accuracy of different methods used to establish the entry points of the MPFL femoral tunnel. The results of the cadaveric study indicated that the sulcus localization method enabled accurate positioning of the MPFL femoral tunnel. Thus, we applied this localization method in the clinical study, after obtaining ethical approval from our institutional review board.

### Cadaveric study

Twelve fresh-frozen, nonpaired human cadaveric knees were obtained from Shanghai Jiaotong University of Medicine who permitted the use of those specimens in medical research, and written consent was also obtained from next of kin to use these specimens in the present study. There were seven left knees and five right knees (eight females and four males), and the mean age at the time of death was 55.1 ± 16.2 years (range 23–75 years). Each specimen was intact from mid-femur to mid-tibia, with no severe macroscopic degenerative or traumatic changes. All specimens were kept frozen at − 25 °C, and were then thawed overnight at room temperature before dissection.

In this part, we aimed to compare the accuracy of the positioning of the MPFL femoral tunnel entry points using the midpoint, fluoroscopic, and sulcus localization methods. All operations were carried out by one surgeon, and the MPFL femoral tunnel entry points were made as described below.

1. Midpoint localization [7, 8, 12]

**Fig. 1:**
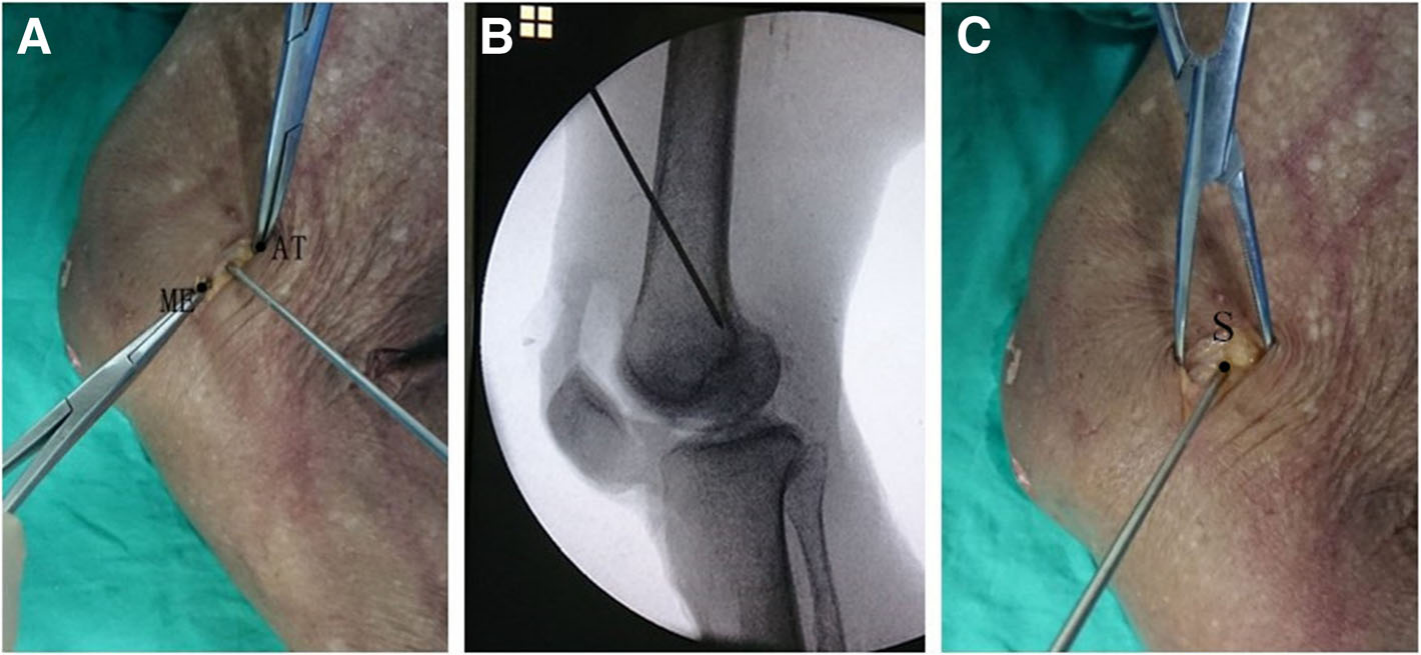
Determination of the femoral tunnel entry point of the medial patellofemoral ligament using three different localization methods. **a** Midpoint localization. **b** Fluoroscopic localization. **c** Sulcus localization. ME: medial epicondyle; AT: adductor tubercle; S: sulcus

An approximately 2 cm longitudinal incision was made over the medial femoral condyle. The subcutaneous tissue was then bluntly dissected to enable palpation of the AT and the ME, which were determined as the largest protruding osseous on the medial femur. A green metal pin was pressed into the bone at the midpoint between the AT and the ME, as determined via visual inspection (Fig. 1a). It took less than 1 min from skin incision to the time when the MPFL femoral tunnel entry point was established.

2. Fluoroscopic localization [4, 8]

An approximately 2 cm longitudinal incision was made over the medial femoral condyle. An assistant operated a mini C-arm (Philips Medical Systems; Netherlands B.V) to obtain a true lateral radiograph, with the posterior portions of the lateral and medial femoral condyles superimposed on each other. A guidewire was then used to locate the MPFL femoral tunnel entry point. To avoid damaging the native MPFL attachment and geography of the medial femur, the guidewire was not inserted into the bone. The guide wire was positioned anterior to the extension of the most posterior portion of the posterior femoral cortex, and between two horizontal lines extending from the cortex-metaphyseal flare junction and the most posterior aspect of Blumensaat’s line. A red pin was then pressed into the bone at the position of the guide wire (Fig. 1b). It took around 10 min from skin incision to the time when the MPFL femoral tunnel entry point was established.

3. Sulcus localization

**Fig. 2:**
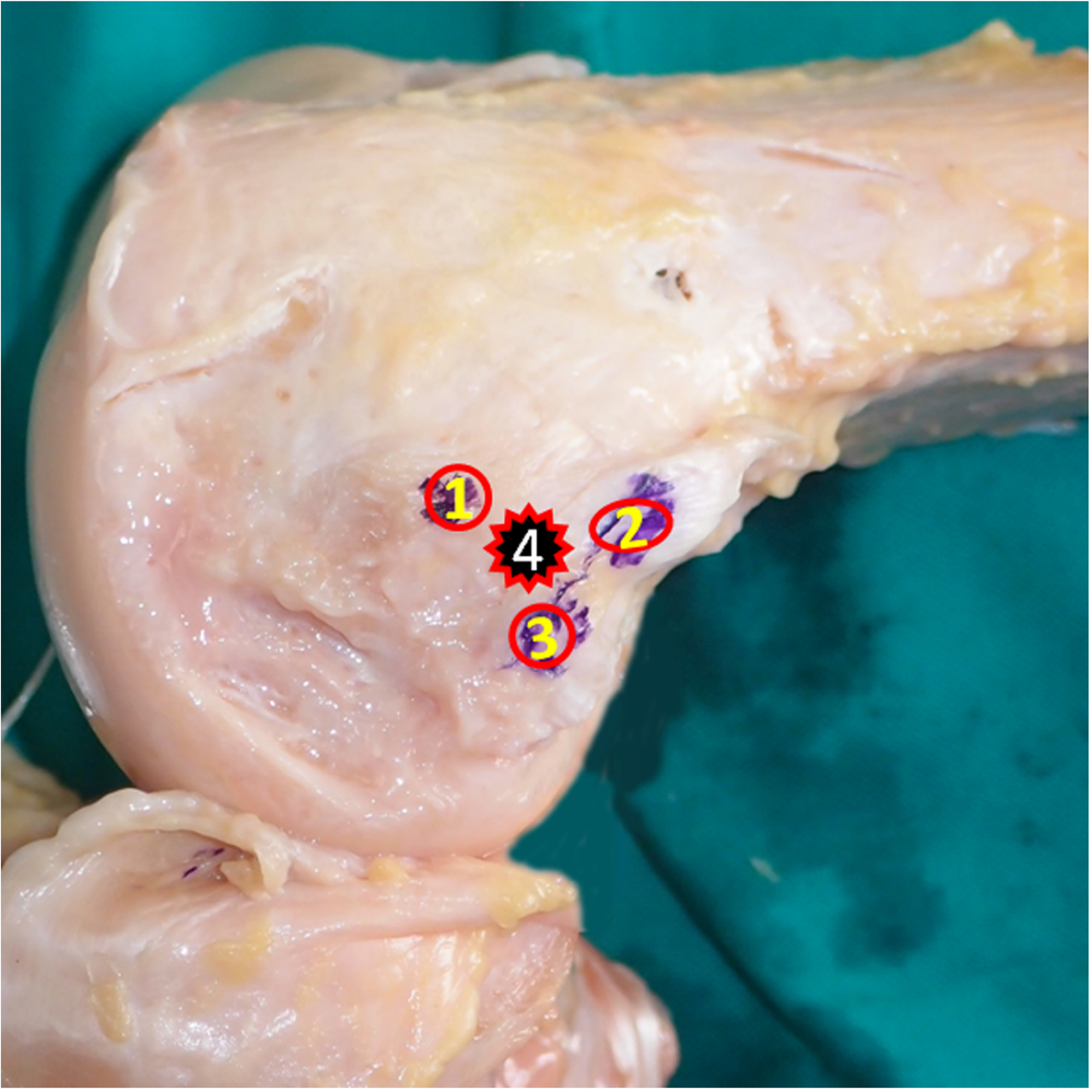
Relative position of the sulcus and three bony prominences in the medial aspect of the knee. 1: the medial epicondyle; 2: the adductor tubercle; 3: the gastrocnemius tubercle; 4: the sulcus

Though this method is called sulcus localization, it is actually three-prominence-dependent localization. During operation, through the approximately 2-cm skin incision, all the soft tissue layers over the three bony prominences were incised longitudinally to expose the bone surface of the medial femoral condyle. Though the soft tissue window is small, direct touch of the three bony prominences were realized. Technically, the AT was detected with finger sliding in a proximal-to-distal way along the medial aspect of the femur. With the AT as a reference, the ME and GT were located at the distal-anterior and distal-posterior side of it with finger sliding and touching. When the three prominences were localized, the central position among the three prominences, which was always a short longitudinal sulcus or dent with a size of approximately 1 cm in most cases, could be easily located with finger-touching. In rare case when the sulcus or dent is not so clearly defined because of a too-shallow dent, the mid-point among the three prominences could still be easily located (Fig. 2). After palpation of the sulcus, a black pin was pressed into it as close to is center as possible (Fig. 1c). It took less than 1 min from skin incision to the time when the MPFL femoral tunnel entry point was established.

**Fig. 3:**
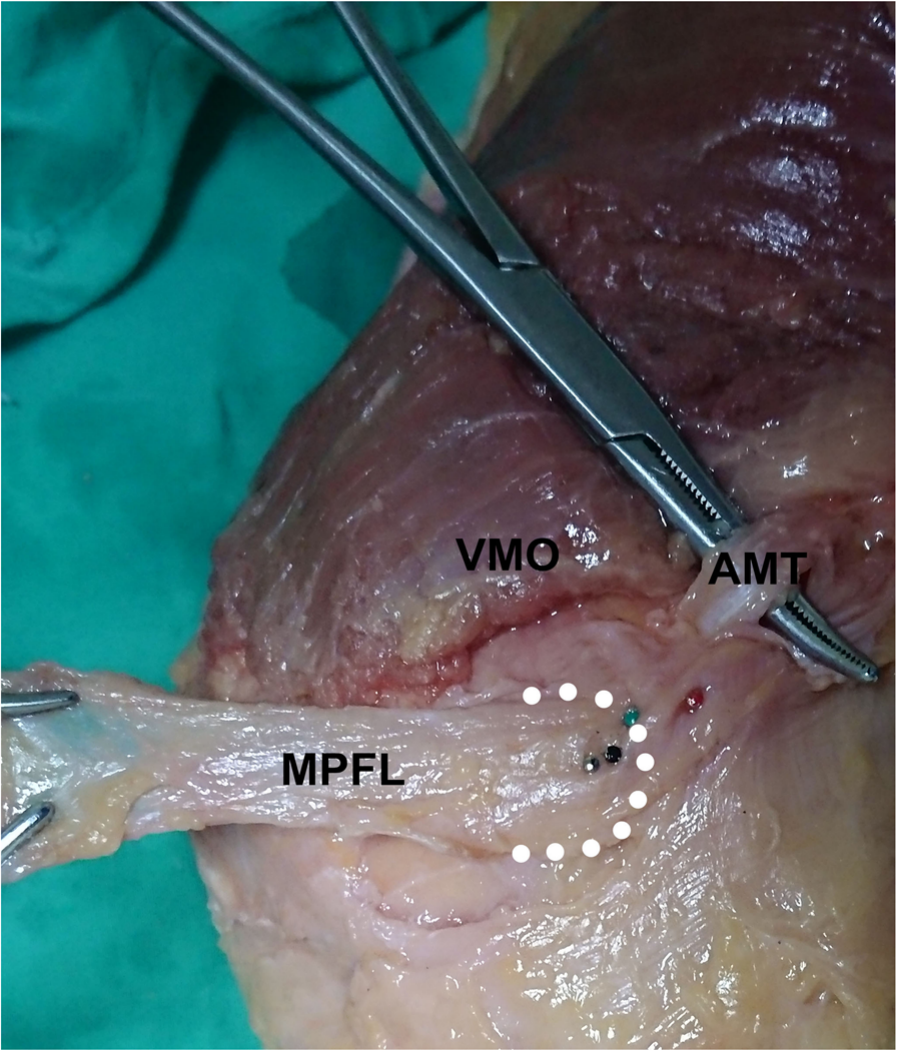
A representative picture showing the medial aspect of a right cadaveric knee. The white circles depict the medial patellofemoral ligament (MPFL) femoral attachment and the grey pin marks the center of the MPFL femoral attachment. The green, red, and black pins represent the femoral tunnel entry points established using the midpoint, fluoroscopic, and sulcus localization methods, respectively. AMT: adductor magnus tendon; VMO: vastus medialis obliquus

The order in which these three localization methods were applied to the cadaveric knees was varied in an even manner to prevent subjective bias from being induced by the prior method (Table 1). After establishment of the MPFL femoral tunnel entry points, further dissections were performed to identify the native MPFL attachment on the femoral side. The skin and subcutaneous tissue were incised at the midline of the knee. The skin and subcutaneous tissue on the medial side of the knee were then removed, and the proximal edge of the MPFL was released from the vastus medialis obliquus. The MPFL was released from its patellar insertion, and careful dissection was performed to expose the femoral insertion. The femoral attachment of the MPFL was defined as the region from which its fibers originated. That attachment was exactly located in the sulcus region, which could be palpated by finger touching. A grey metal pin was pressed into the bone to mark the center of the femoral attachment of the MPFL, and this served as the reference for the following analysis (Fig. 3).

**Table 1:** Order in which different localization methods were applied to the cadaveric specimens

Specimen	Order
1 + 6n	ML → FL → SL
2 + 6n	ML → SL → FL
3 + 6n	SL → ML → FL
4 + 6n	SL → FL → ML
5 + 6n	FL → ML → SL
6 + 6n	FL → SL → ML

*n* = 0,1; *ML* Midpoint localization, *FL* fluoroscopic localization, *SL* sulcus localization

**Fig. 4:**
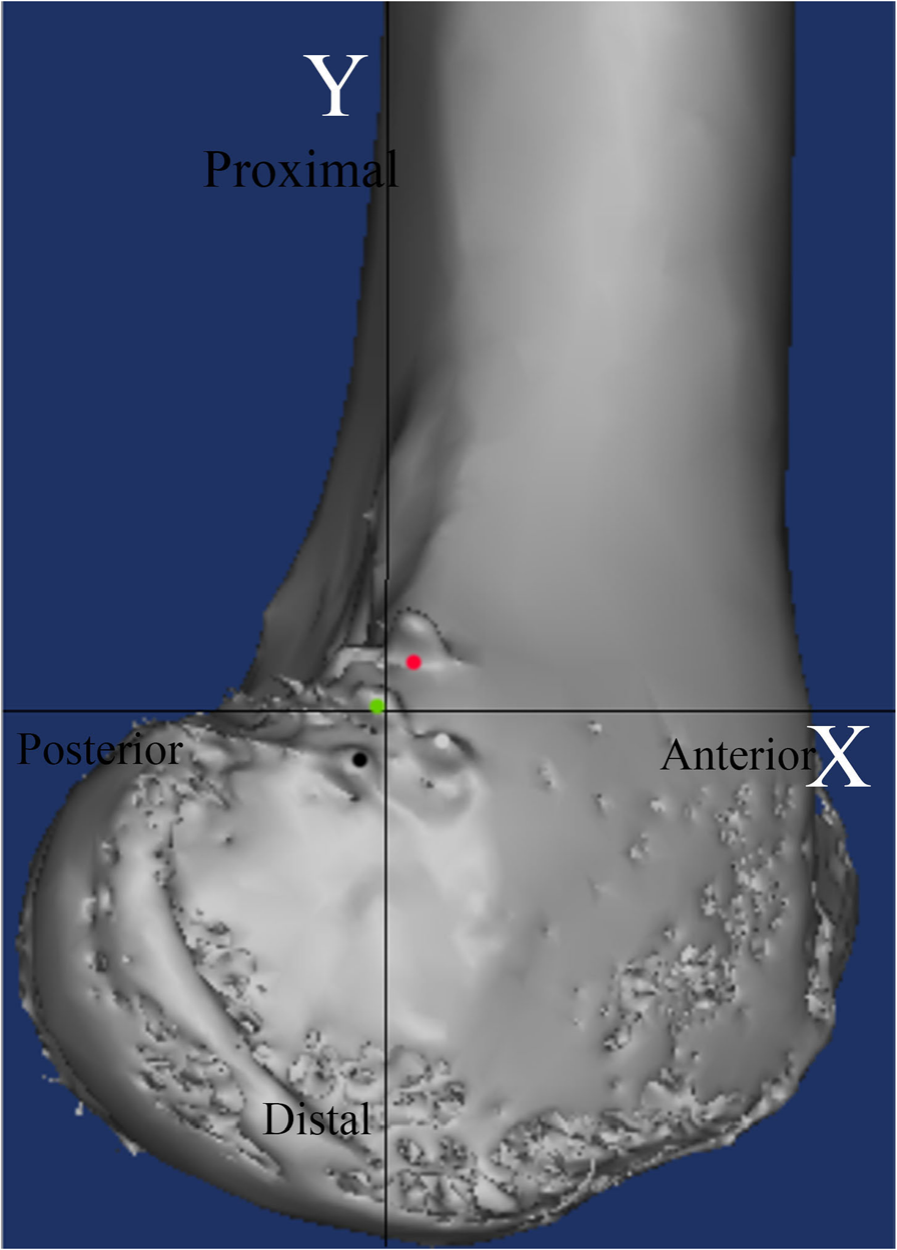
A representative computed tomography image showing the three medial patellofemoral ligament femoral tunnel entry points established using different localization methods. The grey circle represents the center of the native medial patellofemoral ligament attachment in a cadaveric knee. The black, red, green circles represent the centers of the femoral tunnel entry points established by the sulcus, fluoroscopic, and midpoint localization methods, respectively

Computed tomography (CT) (Siemens Medical System; Erlangen, Germany) was performed for every specimen, and Digital Imaging and Communications in Medicine (DICOM) data of 1-mm axial plane slices obtained from the CT scans were reconstructed into three-dimensional (3-D) constructs using Mimics imaging processing software (version 15.0 and MedCAD module; Materialise N.V., Belgium). A true lateral view of the knee was obtained using this dedicated software in transparent mode to perfectly coincide the posterior portion of the medial femoral condyle and the lateral femoral condyle to avoid introducing rotation or inclination errors [18]. A two-dimensional coordinate system was introduced by defining the Y-axis as the extension of the most posterior portion of the posterior femoral cortex (with positive values assigned to points located anterior to the Y-axis), and the X-axis as a horizontal line intersecting the point of contact between the posterior femoral condyle and the posterior cortex (with positive values assigned to points located proximal to the X-axis) [4, 21, 22]. The position of the center of the MPFL attachment was determined using the coordinate system, and the straight-line distance was measured from the center of the MPFL attachment to the centers of the femoral tunnel entry points established using each of the three localization methods (Fig. 4). To avoid interobserver error, the same observer performed each data measurement three times with a 2-week interval between measurements; the observer was blinded to the previous measurements.

### Clinical study

The clinical study comprised a retrospective analysis of prospectively collected data. From June 2017 to December 2017, 53 patients with recurrent patellar dislocation were assessed for enrollment in the present study. The inclusion criteria were: (1) more than two episodes of patellar dislocation, or one episode of patellar dislocation plus multiple episodes of instability, (2) MPFL injury determined on magnetic resonance imaging. The exclusion criteria were: (1) previous patellofemoral joint surgery, (2) other concomitant ligament injury of the ipsilateral knee, (3) fracture of the patellar, or the medial or lateral femoral condyles, (4) open physes or the presence of patellofemoral joint degenerative arthropathy. The study group included 60 knees from 39 females (42knees) and 14 males (18knees); there were 33 left knees and 26 right knees. The mean patient age at the time of surgery was 19.4 ± 4.6 years (range 15–41 years), and the mean age at the time of the first patellar dislocation was 18.0 ± 4.6 years (range 11–32 years). Ethics committee approval was obtained for the present study.

Two experienced surgeons performed the surgeries in all patients using regional or general anesthesia. The anterior half of the peroneus longus tendon was used for the MPFL reconstruction in all patients. A single patellar tunnel was first created in an oblique manner to avoid patellar fracture. An approximately 2 cm longitudinal incision was made over the ME, and the subcutaneous tissue was bluntly dissected to enable palpation of the sulcus as we above-mentioned in the cadaveric study. A guidewire was initially used to drill at the sulcus, and then the guidewire was over-drilled with a drill width corresponding to the width of the tendon. For both surgeons, it took less than 1 min from skin incision to the time when the MPFL femoral tunnel was established. The femoral tunnel was made from distal-medial to proximal-lateral and exited from the lateral side of the femoral shaft. The graft was passed through the patellar tunnel from medially to laterally, and was pulled back over the anterior surface of the patella. Lateral release was performed to pull the patella medially with the graft. Both ends of the graft were then pulled into the femoral tunnel and the graft was fixed with an 8-mm nonmetal interference screw. Finally, a modified Fulkerson osteotomy was performed to transfer the tibial tubercle in case with a tibial tubercle–trochlear groove distance of greater than 15 mm.

**Fig. 5:**
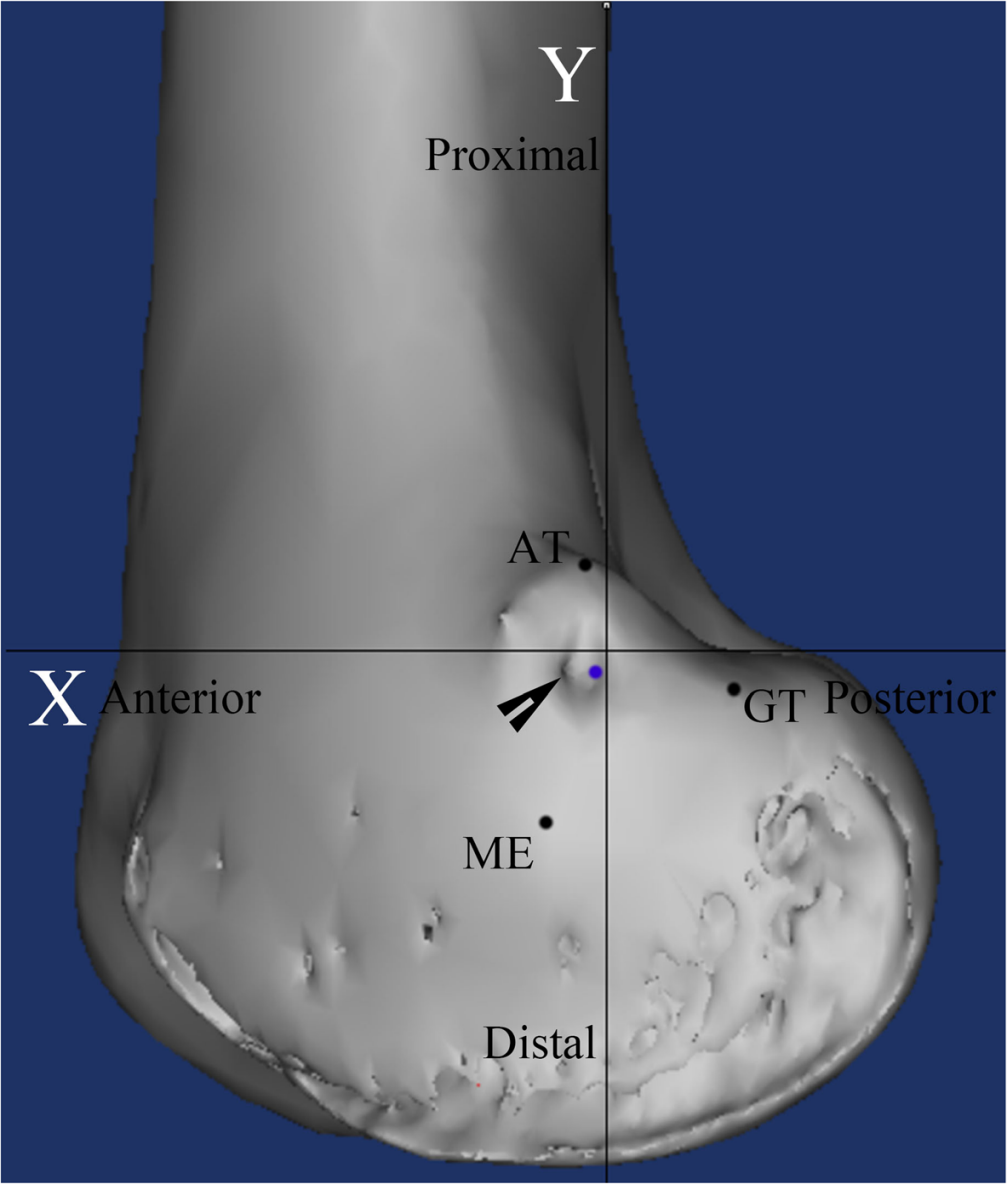
A representative computed tomography image showing the assessment of the accuracy of the medial patellofemoral ligament femoral tunnel. The blue circle indicates Schöttle point, and the black arrow indicates the center of the medial patellofemoral ligament femoral tunnel established using the sulcus localization method. AT: adductor tubercle; ME: medial epicondyle; GT: gastrocnemius tubercle

Every patient underwent CT (Siemens Medical System; Erlangen, Germany) immediately after the surgery, and DICOM data of 1-mm axial plane slices obtained by CT were reconstructed into 3-D constructs with Mimics imaging processing software (version 15.0 and MedCAD module; Materialise N.V., Belgium). The center of the femoral tunnel was defined as the midpoint of the 8 mm nonmetal interference screw. The apices of the AT, ME, and GT were determined as the furthest protruding points on the 3-D construct. After identification of the four abovementioned points, a true lateral view of the knee was obtained. A two-dimensional coordinate system was introduced in the same way as in the cadaveric study. The accuracy of femoral tunnel placement was assessed using established radiographic criteria [4]: Schöttle’s point was defined as (1.3,-2.5) [4], and the straight-line distance from the center of the femoral tunnel to Schottle’s point was measured. We defined accurate placement as positioning of the femoral tunnel less than 5 mm from Schöttle’s point, as previous studies reported that malpositioning of the femoral tunnel by as little as 5 mm deviation from the native MPFL attachment significantly altered the graft isometry and patellofemoral force distribution [3, 10]. The distance from the femoral tunnel to each osseous prominence was also measured in both the posterior-anterior and distal-proximal directions (Fig. 5). Data were gathered in the same way as in the cadaveric study.

Statistical analyses were performed using SPSS software (version 14, SPSS, Chicago, Illinois). In the cadaveric study, analysis of variance was first performed using the F test to detect whether there was a significant difference between the three localization methods regarding the distance from the native MPFL attachment to the femoral tunnel entry point. When the F test indicated the existence of significant differences, the Newman-Keuls q test was used to perform multiple comparisons between the results from different localization methods. The significance level was set at .05 for the analysis of variance analyses.

## Results

### Cadaveric study

The mean distances from the center of the native MPFL attachment to the entry point of the femoral tunnel were 4.2 ± 1.0 mm (range 2.4–5.6 mm), 4.4 ± 1.4 mm (range 1.8–6.6 mm), and 2.9 ± 0.8 mm (range 1.9–4.4 mm) using the midpoint, fluoroscopic, sulcus localization methods, respectively. Statistically significant differences were observed in the results between the midpoint and sulcus localization methods, and between the fluoroscopic and sulcus localization methods (*P* ≤ .05). No statistically significant difference was observed between the midpoint and fluoroscopic localization methods (n.s.).

**Fig. 6:**
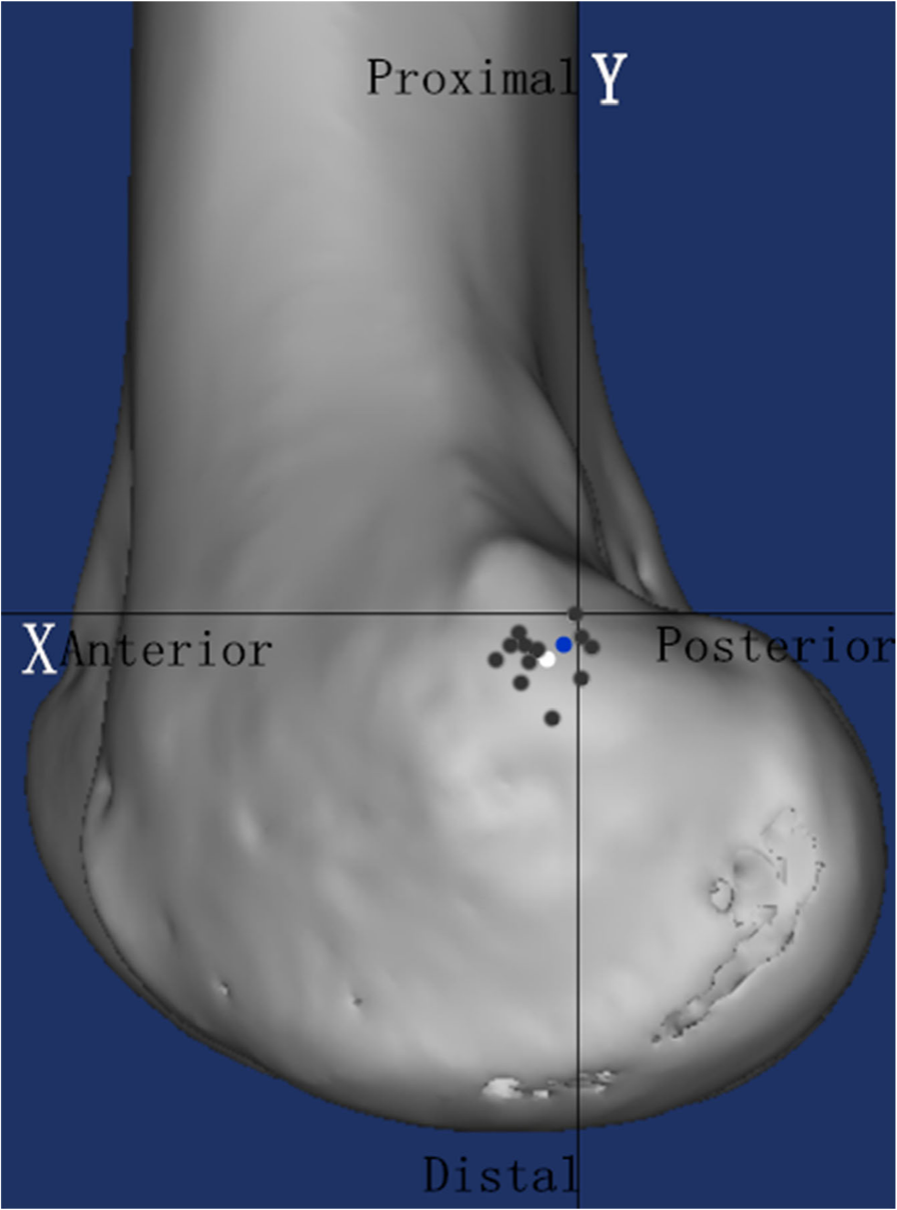
A representative computed tomography image showing the distribution of the locations of all the medial patellofemoral ligament attachment. The blue circle indicates the location of Schöttle point, the black circles indicate the centers of the native medial patellofemoral ligament attachments of all 12 specimens, and the white circle represents the mean position of all the medial patellofemoral ligament attachments

Based on the CT measurement, the mean MPFL attachment was located 2.0 ± 1.8 mm (range − 0.7-4.7 mm) anterior to the most posterior portion of the posterior femoral cortex, and 3.4 ± 1.7 mm (range 0.7–7.5 mm) distal to a horizontal line intersecting the contact of the posterior femoral condyle with the posterior cortex. These results were close to the location of Schöttle’s point reported in an original radiographic study (Fig. 6). Therefore, we considered that Schöttle’s point could be used as a radiographic reference to assess the accuracy of the sulcus localization method to establish the MPFL femoral tunnel in the clinical study.

### Clinical study

**Fig. 7:**
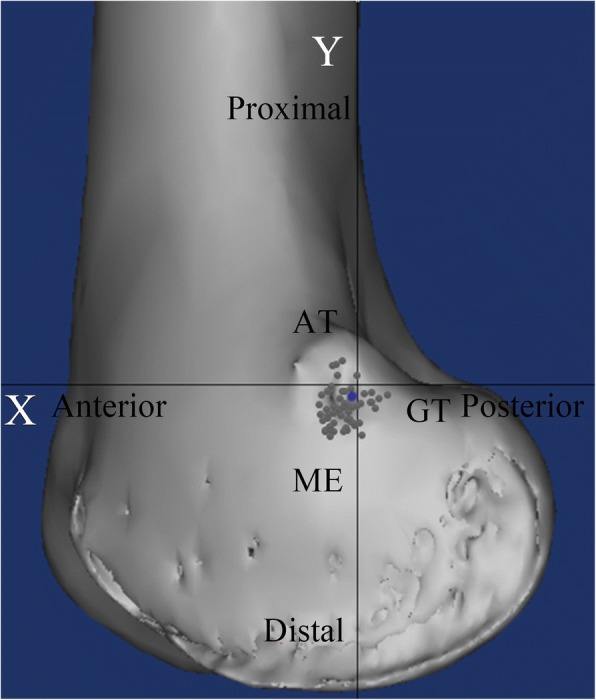
A representative computed tomography image showing the distribution of the locations of all the medial patellofemoral ligament femoral tunnels. The blue circle indicates the location of Schöttle point, the black circle indicates the centers of the medial patellofemoral ligament femoral tunnels in all 60 reconstructed knees. AT: adductor tubercle; ME: medial epicondyle; GT: gastrocnemius tubercle

Quantitative evaluation of the postoperatively reconstructed 3-D images revealed that the mean distance from Schöttle’s point to the center of the MPFL femoral tunnel established using the sulcus localization method was 3.5 ± 1.5 mm (range 0.4–6.1 mm). The center of the MPFL femoral tunnel was located less than 5 mm from Schöttle’s point in 49 of 60 knees (82%). The femoral tunnel was located a mean distance of 9.5 ± 2.5 mm (range 2.8–4.9 mm) distal and 0.1 ± 2.0 mm (range − 4.7-4.7 mm) posterior to the AT, 1.0 ± 2.5 mm (range − 5.1-5.6 mm) distal and 9.5 ± 2.5 mm (range 3.7–14.8 mm) anterior to the GT, and 9.6 ± 2.3 mm (range 4.3–17.5 mm) proximal and 7.5 ± 2.0 mm (range 2.5–12.3 mm) posterior to the ME (Fig. 7).

## Discussion

The present study is the first to compare the accuracy of three different localization methods to establish the MPFL femoral tunnel. The results of our cadaveric study showed that the sulcus localization method was more accurate than both the midpoint and fluoroscopic localization methods. The sulcus localization method accommodates for individual variability and best mimics anatomic reconstruction of the MPFL. The time taken to establish the MPFL femoral tunnel using the sulcus localization method was comparable with that required for the midpoint localization method, and was shorter than that required for the fluoroscopic localization method; thus, the fluoroscopic method has the disadvantages of a prolonged surgery time and irradiation exposure.

In anatomic reconstruction of the MPFL, the aim is to optimally restore normal patellofemoral kinematics by performing accurate tunnel placement, especially on the femoral side [1, 3, 5, 9, 10]. Positioning the femoral tunnel too far proximally reportedly causes significantly increased graft tension during knee flexion, leading to early arthritis, graft rupture, or graft failure; conversely, positioning the femoral tunnel too far distally reportedly results in a nontensioned graft that is insufficient as a medial restraint [23]. Although various localization methods have been well described to guide the tunnel placement based on intraoperative fluoroscopy [1, 4, 8, 16, 17] or merely estimating approximate distance from osseous prominences [2, 7, 8, 12, 18], nonanatomic femoral tunnel placement is still prevalent, causing postoperative complications [8, 11]. Hence, there is a need for a reproducible and accurate localization method for determining the placement of the MPFL femoral tunnel.

Recent research has reported that there is a palpable sulcus at the femoral attachment of the MPFL. A previous study reported that this sulcus was the anatomic attachment of the MPFL in all 50 dissected specimens [20], and this was confirmed in other studies [1, 5, 21]. These findings provide strong theoretical support for using a sulcus localization method to accurately position the MPFL femoral tunnel.

In the present study, the mean distance from the center of the native MPFL attachment to the femoral tunnel entry point established using fluoroscopic guidance was comparable to the results reported in a previous study [22]. Obtaining a true lateral view of the knee can be very difficult in the setting of practical surgeries, and a minor deviation from true lateral knee radiograph could significantly alter the femoral tunnel placement [17, 21, 24]. Ziegler et al. [21] strongly argued against the use of radiographic reference points. They looked at how aberration from a true lateral radiograph altered relationship between the native MPFL attachment and the fluoroscopy-guided tunnel placement. The results of their study showed that a positioning error as little as 5° off-axis from true lateral radiograph could significantly increase the distance from the femoral tunnel to the native MPFL attachment, and that even a 2.5° positioning error caused MPFL tunnel malpositioning of more than 5 mm. Although the present study found this deviation was minor, this could also introduce an inherent error when using the fluoroscopic localization method.

The midpoint localization method also resulted in a potentially deviated femoral tunnel for several reasons. First, there is variability in the MPFL femoral attachment relative to the osseous prominences [2, 13–15, 18, 20, 21, 25]. The MPFL attachment is generally proximal and posterior to the ME, and distal to the AT. However, the anterior-posterior relationship between the AT and the MPFL attachment varies, with previous studies reporting that the MPFL attachment was found to be posterior to the AT [2, 21], just distal to the AT [18], and anterior to the AT [14, 15, 25]; these differences may be due to individual variability [21]. The location of the osseous prominences varies as a consequence of individual weight-bearing activity [1], so the anterior-posterior relationship between the AT and the MPFL attachment may not be constant. Furthermore, recent studies have reported that the MPFL femoral attachment was closer to the AT than the ME [14, 15, 21]; drilling the femoral tunnel at the midpoint between the AT and ME typically places the tunnel anterior to the AT, and at an equal distance from the AT and the ME. Second, the AT and the ME are reportedly not easily identified [7]. Similarly, we found that the osseous prominences in some of the specimens were not easily located via palpation, increasing the possibility of establishing a deviated tunnel.

The quantitative evaluation of postoperative images in our clinical study revealed that using the sulcus localization method to establish the MPFL femoral tunnels demonstrated a high degree of accuracy compared with previous studies [7, 19]: 82% of the tunnels were located inside the defined area (less than 5 mm from Schöttle’s point), the most malpositioned tunnel was only 6.1 mm from Schöttle’s point, and the distribution of all the femoral tunnels (Fig. 6) was quite similar to the position of the MPFL attachment reported in a recent systematic review that assessed 57 studies reporting the anatomy of the MPFL on the femoral side [6]. In contrast to the present study, a previous study using the midpoint localization method reported that 31% of the tunnels were located more than 7 mm from Schöttle’s point on postoperative radiographic evaluation [7], another study reported a malposition rate of 64% (with tunnel placement regarded as accurate if the entry point was positioned within 9 mm from Schöttle’s point) without describing a specific localization method [19], and another study reported that 28% of the tunnels were nonanatomic (with the cutoff set at 10 mm from Schöttle’s point) [11].

Recent studies evaluating the validity of radiographic reference points showed great variability, even with a true lateral radiograph [1, 4, 16, 17, 21, 22]. The native MPFL attachment center reportedly ranges from 8.8 mm anterior to 1.3 mm posterior to the posterior femoral cortex line, and 2.5 mm to 6.4 mm distal to the horizontal line intersecting the contact of the posterior femoral condyle with the posterior cortex [1, 4, 16, 17, 21, 22]. This great discrepancy could be partially explained by the relatively limited number of specimens used in these previous studies and partially by the varying definitions of the posterior cortex line, which would introduce errors in both the anterior-posterior and proximal-distal directions. The definition of the posterior cortex line in different studies was reported as “a fixed point along the cortex” [1], and “a continuation of the posterior femoral cortex” [17]. After narrowing the definition of the posterior cortex line to “the most posterior portion of the posterior femoral cortex”, which was confirmed by Redfern et al. [22] by their communication with the original authors [4], the remaining radiographic reference points were all located in the region less than 2.5 mm from Schöttle’spoint: Ziegler et al. [21] reported that the mean MPFL attachment was 2.3 mm anterior to the most posterior portion of the posterior femoral cortex and 1.6 mm proximal to the horizontal line through the most posterior aspect of Blumensaat’s line, and Redfern et al. [22] reported that the radiographic point was located only 0.8 mm posterior to Schöttle’s point. Similarly, the results of our cadaveric study put the radiographic point at 0.7 mm anterior, and 0.9 mm distal to Schöttle’s point. As both inter- and intraobserver errors may have contributed to the slight deviation in the MPFL attachment position relative to radiographic reference lines, we considered that Schöttle’s point still serves as a useful reference point with which to postoperatively assess the accuracy of MPFL femoral tunnel placement.

Two previous studies investigated the clinical effect of malpositioned femoral tunnels [7, 19], and reported that a tunnel that was malpositioned by 7 mm or even more than 9 mm did not affect the outcome at final follow-up; a correlation between the tunnel placement and clinical outcomes was only reported after the cutoff was increased to 10 mm [11]. These outcomes run opposite to the results of biomechanical studies that report a significant effect of a slightly deviated femoral tunnel on the graft length change patterns, medial patellofemoral contact pressures, resultant chondropathy, and recurrence of patellofemoral instability [1, 9, 10]. Possible factors associated with these conflicting results include the duration of follow-up, degree and direction of malpositioned tunnels, sensitivity of outcome measurements, other abnormalities of an unstable patellofemoral joint, and other concomitant surgical procedures and properties of the graft [7, 24, 26]. Another main factor that may contribute to these conflicting results is that the femoral attachment area of both the native MPFL and the reconstructed graft is wide [24], while the biomechanical studies measured length change patterns based on single points [1, 9]. Thus, deviation could be somewhat acceptable within a certain range, as long as the functional portion of the graft is within the native MPFL attachment area [24]. Accordingly, significant differences that were seen between different localization methods in the present cadaveric study might not necessarily lead to significantly different clinical outcomes in actual surgeries. We considered that the midpoint or fluoroscopic localization methods may be able to give the same satisfactory clinical outcomes as the sulcus localization method (provided that surgeons could use it appropriately), and that the 5-mm cutoff set in the present clinical study might have been too rigid. Further studies with more precise designs comparing the clinical outcomes resulting from femoral tunnel placements with varying degrees of deviation from the native MPFL attachment are warranted, and a tolerable range of deviation should be used to guide the MPFL femoral tunnel placement.

The present study has some limitations. First, the clinical study employed Schöttle’s point as the reference for judging the accuracy of MPFL femoral tunnel placement, rather than using the center of the native MPFL attachment in each patient. As we mentioned, the MPFL attachment in specific individuals could deviated from Schöttle’s point, which might introduce a systematic error to the way in which we assessed the accuracy of MPFL femoral tunnel placement in the clinical study. However, ethical issues prevented the performance of further dissection to identify the native MPFL attachment and mark its center in patients, let alone in the setting of a tear of the MPFL off the femoral side [3]. Second, cadavers used in the present come from a normal population yet MPFL comes from a population with patellar instability of whom all have anatomical abnormalities in bone morphology to a varying degree. The dysplasia affects the whole distal femur and in the more severe cases, there is no sulcus where the femoral origin of the MPFL should be; and it also affects the soft tissues as well. Thus, inferring clinical decision making from a normal population is likely to be inaccurate. In cases when patients present severe patellofemoral abnormalities, it’s recommended to locate the MPFL femoral tunnel according to practical situations during the surgery. Third, the generalizability of the sulcus localization method remains unknown. In the present study, palpation of the sulcus was done by two experienced surgeons. The soft tissues around the bony sulcus might influence the accurate determination of this landmark, and an approximately 2 cm opening might be too small to be used as an approach window. Hence, it is doubtful whether junior surgeons could establish accurate MPFL femoral tunnel placement using the sulcus localization method via a mini incision. Moreover, as several previous showed that the post-operative outcomes did not correlate with the femoral tunnel position with respect to Schöttle’s point [7, 19, 27], it is necessary to correlate the radiological findings with the clinical outcomes, which hasn’t been done in the present study. Finally, the present cadaveric study was limited by the small sample size.

## Conclusions

In a population with normal knees the femoral origin of the MPFL can be accurately identified intra-operatively by feeling for a sulcus. The sulcus localization method can achieve accurate placement of the MPFL femoral tunnel. Considering its accuracy and operability, the sulcus localization method might be generalized for surgeons.

## Abbreviations

AT: Adductor tubercle
CT: Computed tomography
GT: Gastrocnemius tubercle
ME: Medial epicondyle
MPFL: Medial patellofemoral ligament
